# Understanding nonlinear and synergistic effects of the built environment on urban vibrancy in metro station areas

**DOI:** 10.1186/s44147-023-00182-z

**Published:** 2023-03-09

**Authors:** Jiandong Peng, Yiwen Hu, Chao Liang, Qiuyu Wan, Qi Dai, Hong Yang

**Affiliations:** 1 School of Urban Design, Wuhan University, Wuhan, China; 2 Guangdong Guodi Institute of Resources and Environment, Guangzhou, China; 3 Wuhan Planning & Design Institute (Wuhan Transportation Development Strategy Institute), Wuhan, China

**Keywords:** Urban vibrancy, TOD, Built environment, Gradient boosting decision tree, Nonlinear effects, Synergistic effects, Different times

## Abstract

Transit-oriented development (TOD) has long been recognized as a significant model for prospering urban vibrancy. However, most studies on TOD and urban vibrancy do not consider temporal differences or the nonlinear effects involved. This study applies the gradient boosting decision tree (GBDT) model to metro station areas in Wuhan to explore the nonlinear and synergistic effects of the built-environment features on urban vibrancy during different times. The results show that (1) the effects of the built-environment features on the vibrancy around metro stations differ over time; (2) the most critical features affecting vibrancy are leisure facilities, floor area ratio, commercial facilities, and enterprises; (3) there are approximately linear or complex nonlinear relationships between the built-environment features and the vibrancy; and (4) the synergistic effects suggest that multimodal is more effective at leisure-dominated stations, high-density development is more effective at commercial-dominated stations, and mixed development is more effective at employment-oriented stations. The findings suggest improved planning recommendations for the organization of rail transport to improve the vibrancy of metro station areas.

**Supplementary Information:**

The online version contains supplementary material available at 10.1186/s44147-023-00182-z.

## Introduction

Building vibrant urban spaces promotes human activities and interaction [1], which are crucial for cities to remain resilient and to attract more human resources and economic capital [2]. This concept has been a heated topic of discussion in urban geography, and urban and rural planning research and practice. In China, with the great economic achievements of rapid urbanization since the Reform and Opening Up, urban problems such as “social isolation,” “ghost towns,” and “urban decay” have also occurred, which resulted in the decline and dissolution of urban vibrancy [3, 4]. In particular, due to the 2019 COVID-19 pandemic, people’s ability and willingness to engage in economic and social activities have been dramatically reduced [5]. Transit-oriented development (TOD) has long been recognized as an effective policy for promoting urban vibrancy [6]. The TOD concept was introduced by Calthorpe [7] as a pedestrianized urban area with public transportation as the pivot and integrated development to address the unrestricted urban sprawl. Its promotion not only boosts public transportation ridership and alleviates, but also creates more activity and community life by allowing more people to live closer to each other [8, 9]. However, since people fear that crowded public transport systems would increase the risk of viral infection, human activities around metro stations have declined most dramatically [10]. As the epidemic gradually abates, it is crucial to explore the factors influencing urban vibrancy around metro stations to promote urban economic recovery, sustainable mobility, and social interaction.

The built environment has long been considered a significant factor influencing urban vibrancy, mainly in terms of density, diversity, design, destination accessibility, and distance to transit (5Ds) [11, 12]. It has been shown that compact mixed-use development and a good pedestrian environment are essential ways of promoting urban vibrancy [13, 14]. Station areas, developed under TOD principles, are usually characterized by multidimensional features like high density, mixed land use, pedestrian friendliness, a full range of facilities, and easy access to public transport, making TOD areas a vibrant part of everyday life [15, 16]. Furthermore, residents’ daily mobility and congregations exhibit high spatial and temporal regularity, as people often travel to a place with specific intentions [17]. Therefore, the extent to which the environment influences human activities may also change over time [18, 19]. However, in the existing studies on the built-environment features and urban vibrancy in public transportation areas [20], there is a bias toward comparing spatial differences and less focus on temporal differences. Therefore, exploring the relationship between the built-environment features and the vibrancy around metro stations during different times is necessary.

In existing studies of urban vibrancy and built-environment features, a linear relationship is often assumed, with ordinary least squares or geographically weighted regression used [12, 21, 22]. These studies provide a solid basis for understanding the relationship between the built-environment features and urban vibrancy but ignore the possible nonlinear and synergistic effects of the two [23]. In recent years, scholars have gradually adopted machine learning to elucidate the more refined nonlinear statistical relationship between them. For example, Yang et al. [8] used the gradient boosting decision tree (GBDT) to demonstrate that there is not only a linear relationship between them but also a nearly flat-curve correlation or a nonlinear association that varies within a certain range. GBDT can also compare the relative importance of the independent variables and provide a basis for the planner’s decision time sequence [24, 25]. Moreover, the effects of the built-environment features of TOD on vibrancy may be moderated by third-party variables. Studies have applied the Shapley additive explanations (SHAP) to interpret GBDT model results and provide “Shapley interaction values” to capture feature interactions at the local level (i.e., per sample) [26, 27]. Interaction is the simultaneous influence of two or more independent variables on the dependent variable, providing more information about how the built environment collaborates to shape urban vibrancy [20].

To fill these gaps, this study uses multi-source big data from 210 metro station areas (MSAs) from Wuhan in 2021. It measures urban vibrancy by metro ridership, smartphone location records, and social media check-ins over four specified time intervals: AM peak (7:00–9:00), midday hours (11:00–13:00), PM peak (17:00–19:00), and night hours (20:00–22:00) on weekdays. Subsequently, using the GBDT and SHAP explanatory models, we determine the relative importance of built-environment features and explore in more detail the nonlinear and synergistic effects of the features on the vibrancy of MSAs during different times. With the above model, we can reveal the differences in the effects of built-environment features on urban vibrancy during different times, (2) identify the nonlinear and synergistic effects of built-environment features on the MSAs’ vibrancy, and (3) suggest improved planning recommendations for the organization of rail transport to promote the vibrancy around metro stations.

This study makes a dual contribution to the literature. First, focusing on the metro station stations, this study enriches the existing literature by providing a comprehensive understanding of the impact of various influences on urban vibrancy, including the built environment, metro station characteristics, socioeconomic factors, and different times. Second, this study examines the effects of individual built-environment features on urban vibrancy and the synergistic effects among features, which is more helpful in guiding transportation planning and related policies around metro stations.

## Methods

### Research design

**Fig. 1:**
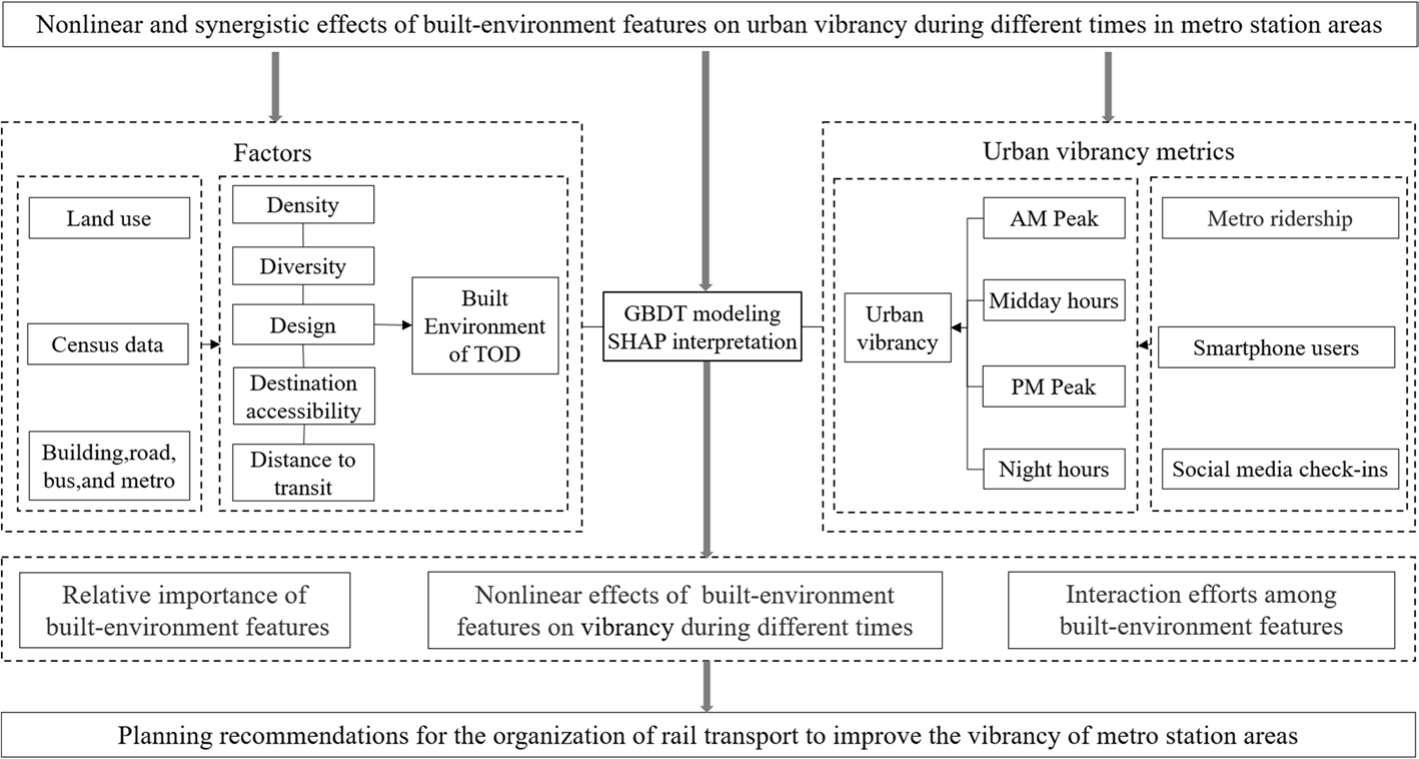
Research design

We conducted the design for this study, as shown in Fig. 1. The design consists of four steps: (1) measuring a composite vibrancy index during different times, (2) constructing built-environment features of TOD based on the “5Ds,” (3) applying the GBDT and introducing the SHAP interpretation to investigate the nonlinear and interaction effects during different times, and (4) proposing an optimization strategy for the MSAs’ vibrancy.

### Study area

**Fig. 2:**
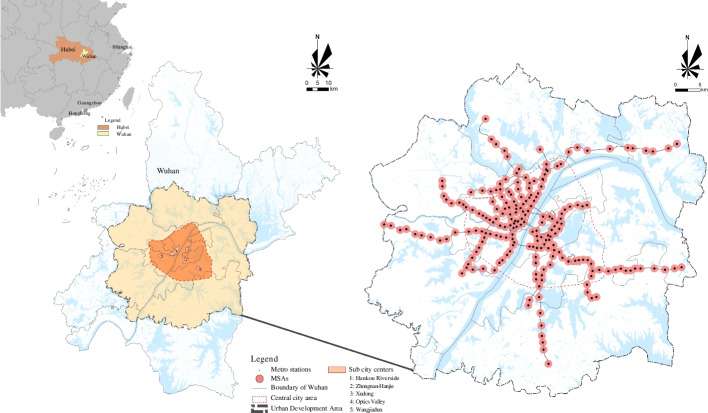
Study area in Wuhan, China

Wuhan is central China’s political, economic, and cultural center, with a permanent population of 13,648,900 in 2021. The city is divided into three parts—Wuchang, Hankou, and Hanyang. The confluence of the Yangtze and Han rivers form an urban pattern of two river meetings and three standing towns. Central and urban development areas mainly bound urban space. At the end of 2019, there was an outbreak of the COVID-19 pandemic in Wuhan, which led to the metro being blocked for 3 months, severely restricting people’s activities around the metro stations. After the unblocking, public transportation was gradually put into everyday use and the city’s vibrancy slowly recovered. By January 2021, Wuhan had opened and operated nine metro lines with 210 stations (transfer stations were not counted repeatedly). Using previous studies as references [28], we used an 800 m radius as the distance threshold for 210 MSAs, as shown in Fig. 2.

### Variables

The dependent variable in this study was the vibrancy of the MSAs. Based on existing research [12, 14], we extracted the combined vibrancy for the three variables of metro traffic, smartphone location records, and social media check-ins during different weekday times using the factor analysis method. Factor analysis is a statistical method proposed by Spearman [29] to extract common factors among some variables that have some correlation. According to Bartlett’s test with KMO=0.713 and *p*≈0.000 in SPSS, we believe the use of factor analysis is appropriate. Principal factor analysis was then employed to extract the composite index with 83.275% of the variance, conveying the primary information of the variables. Table 1 shows the solution of the factor analysis and descriptive statistics of the surface attributes. Metro ridership data was obtained from smart card data for our four specified periods from March 15 to 19, 2021, provided by the Wuhan Institute of Strategic Transport Development. Smartphone location records data were obtained from the Baidu Heat Index for the same periods. Baidu Heat Index records the geographical location of mobile users obtained by the Baidu engine at a certain point in time, projecting the user on a digital map, which can accurately represent the existence of people [8]. Social media check-ins data was obtained from the social media platform Sina Weibo, which collected check-ins for the four time periods on weekdays from March to May 2021.

**Table 1:** The solution of the factor analysis and descriptive statistics of the surface attributes

Indicators	Descriptive statistics			Factor weights	Communalities	
	*N*	Mean	Std		Initial	Extracted
Ln (metro ridership)	840	5.836	1.338	0.835	1.000	0.697
Ln (smartphone location records)	840	6.328	0.989	0.947	1.000	0.897
Ln (social media check-ins)	840	1.930	1.869	0.875	1.000	0.765

The built-environment features of the MSAs were constructed according to the 5D principle. The density included the number of permanent residents of MSAs estimated from the population data of the neighboring communities and the floor area ratio (FAR) and average building height calculated from building data. The land use dimension consisted of four indicators: the land use mix calculated based on the entropy method, the number of leisure facilities (i.e., sports and recreation, parks, and plazas), commercial facilities (i.e., shopping, restaurants), and enterprises (i.e., companies, businesses) obtained by reclassifying the Gaode POI dataset. At the design level, two spatial design network analysis (sDNA) indicators (NQPDA and DivA) were used to represent the centrality and network detour ratio of the streets using the spatial syntax method [20]. Furthermore, the distance to the city center and sub-centers was chosen to represent the accessibility of the area, as well as the number of bus routes to illustrate transfer capacity. For the characteristics of subway stations, we considered the number of entrances and exits, transfer stations, number of reachable metro stations within 20 min, and opening time. The socioeconomic factor included was the average house price in MSAs, calculated based on second-hand house transaction data for Wuhan in 2020, collected by Chain Home. The descriptive statistics of urban vibrancy and the built-environment features of MSAs are shown in Table 2.

**Table 2:** Descriptive statistics of variables

Dimensions	Sub-dimensions	Variables	Descriptions	Mean	Std.
Urban vibrancy	Integrated vibrancy	During AM peak	Integrated vibrancy from 7 to 9	5.347	1.263
		During midday hours	Integrated vibrancy from 11 to 13	5.264	1.317
		During PM peak	Integrated vibrancy from 17 to 19	5.625	1.400
		During night hours	Integrated vibrancy from 20 to 22	5.068	1.492
Built-environment features of MSAs	Residents	Number of residents	Natural logarithm of the number of permanent residents	8.69	1.58
	Building form	Floor area ratio	$=\frac{Grossfloorarea}{MSAArea}$	1.33	0.85
		Average building height	$=\frac{Grossfloorarea}{Grossbuildingfootprint}$	21.03	8.16
	Land use	Land use mix	An entropy index measuring a mixture of residential, public, commercial, industry, green and other land uses $Landuse=\frac{-\sum_{i=1}^k{P}_{ki}\ln \left({P}_i\right)}{\ln k}$ , where *k* is the type of land use and *P*_*ki*_ is the proportion of type-i land use to the total land area [24]	0.64	0.19
		Number of leisure facilities	Natural logarithm of the number of leisure service POIs	2.52	1.32
		Number of enterprises	Natural logarithm of the number of enterprises’ POIs	4.12	1.22
		Number of commercial facilities	Natural logarithm of the number of commercial service POIs	5.79	1.62
	Street network configuration	Street centrality	An sDNA measure of centrality for street network segments called *NQPDA*	17.20	5.31
		Street network detour ratio	An sDNA measure of the degree to which the actual network deviates from the straightest path called *DivA*	1.33	0.06
	Regional accessibility	Distance to the city center	Distance to the city center of Wuhan (km)	11.970	7.212
		Distance to the sub-center	Minimum distance to the sub-centers of Wuhan (km)	7.228	5.888
	Access to transfer	Number of bus routes	Natural logarithm of the total number of routes for each bus station	4.61	1.38
	Metro station features	Reachable metro stations within 20 min	The number of metro stations that one station can reach within 20 min	39.03	23.56
		Number of entrances and exits	Calculate the number of entrances and exits in each metro station	4.65	2.45
		Transfer station	The number of rail transit lines passing through the station	1.14	0.37
		Opening time	Time since the metro station opened (month)	72.01	44.40
	Socioeconomic	Average housing price	Average housing price for each MSA (K RMB)	17.178	5.473

### Modeling approach

A GBDT model was constructed for this study to analyze better the nonlinear effects of built-environment features on the MSAs’ vibrancy and the differences in the effects [8, 20]. The GBDT generates forecasting models in an ensemble of models, and in this case, a regression tree. The algorithm’s objective is to minimize the loss function [25]. The regression tree is defined as follows:

1 $$F(x)={\sum}_{i=1}^m{f}_m(x)={\sum}_{i=1}^m{\alpha}_{jm}I\left(x;{\varepsilon}_m\right)$$

where the parameter *ε*_*m*_ is denoted as each regression tree *I*(*x*; *ε*_*m*_) in the split position and the mean value of the terminal nodes in the regression tree *α*_*jm*_ is estimated by minimizing the loss function [24]. The optimization process consists of several iterative steps.

First, initialize the weak learner *f*_0_(*x*):

2 $${f}_0(x)={argmin}_{\varepsilon }{\sum}_{i=1}^{\textrm{N}}L\left({y}_i,\varepsilon \right)$$

Secondly, for the number of iterative rounds *m*(*m* = 1, 2, 3, …, *M*), there are:

- For each sample *i*(*i* = 1, 2, 3, …, *N*), we calculated the negative gradient (i.e., residuals) *ε*_*im*_:

3 $${\varepsilon}_{im}=-{\left[\frac{\partial L\left({y}_i,f\left({x}_i\right)\right)}{\partial f\left({x}_i\right)}\right]}_{f(x)={f}_{m-1}(x)}$$

(b) Fit a regression tree to the residuals *ε*_*im*_ and obtain the leaf node region of the *m*th tree *A*_*jm*_ where *j* = 1, 2, 3, …, *J*, i.e., a tree consisting of *J* tree of leaf nodes.
(c) For leaf region *j*, calculate the best-fit value, *ε*_*jm*_:

4 $${\varepsilon}_{jm}=\mathit{\arg}\underset{\varepsilon }{\mathit{\min}}{\sum}_{x_i\in {A}_{jm}}L\left({y}_i,{f}_{m-1}\left({x}_i\right)+\varepsilon \right)$$

(iv) Updating strong learners *f*_*m*_(*x*):

5 $${f}_m(x)={f}_{m-1}(x)+{\sum}_{j=1}^J{\varepsilon}_{jm}I\left(x\in {A}_{jm}\right)$$

Finally, the operation is concluded, and the final learner is obtained

6 $$f(x)={f}_M(x).$$

In this study, we introduced a learning rate factor *ϕ*(0 < *ϕ* ≤ 1) to bind the residual learning outcomes for each regression tree [30].

7 $${f}_m(x)={f}_{m-1}(x)+\phi \cdot {\sum}_{j=1}^J{\varepsilon}_{jm}I\left(x\in {A}_{jm}\right),0<\phi \le 1$$

The modeling process for this study was performed using the GBDT package in Python 3.6. First, we randomly divided the data into two parts: a training set (80%) and a test set (20%). We then applied Huber as the loss function and used 5-fold cross-validation techniques to adjust the seven parameters to obtain the optimal combination of parameters, as shown in Table 3.

**Table 3:** Parameters specified in the model

Hyper-parameters	Descriptions	Optimal hyper-parameters
learning_rate	Iteration speed	0.01
n_estimators	Number of iterations/trees	800
subsample	Subsampling	0.8
min_samples_split	Minimum sample	3
min_samples_leaf	Minimum number of samples	2
max_features	Maximum number of features	log2
max_depth	Maximum tree depth	21

To explain the results of the GBDT, we chose the SHAP model developed by Lundberg and Lee [31]. This technique helps to understand the nonlinear and synergistic relationship between the built-environment features and vibrancy. Inspired by cooperative game theory, SHAP constructs an additive explanatory model in which all features are considered “contributors” [32]. When the model is nonlinear or the input features are not independent, SHAP calculates a weighted average by ranking all the possible features. SHAP interprets the prediction of the model as the sum of the imputed values of each input feature, where the assigned values are the Shapley values [28] calculated according to the following equation:

8 $$g(x)={\phi}_0+\sum\nolimits_{j=1}^M{\phi}_j$$

where *ϕ*_0_ is the constant that explains the model and is the predicted mean of all training samples. Each feature has a corresponding Shapley value, i.e., *ϕ*_*j*_:

9 $${\phi}_j=\sum\nolimits_{S\subseteq \left\{{x}_1,\cdots, {x}_p\right\}\setminus \left\{{x}_j\right\}}\frac{\left|S\right|!\left(p-\left|S\right|-1\right)!}{p!}\left({f}_x\left(S\cup \left\{{x}_j\right\}\right)-{f}_x(S)\right)$$

where {*x*_1_, ⋯, *x*_*p*_} is the set of all input features, *p* is the number of all input features, {*x*_1_, ⋯, *x*_*p*_} ∖ {*x*_*j*_} is the set of all possible input features that do not include{*x*_*j*_}, and *f*_*x*_(*S*) is the set of feature subsets*S* of predictions. The weight $\frac{\left|S\right|!\left(p-\left|S\right|-1\right)!}{p!}$ can be interpreted as follows: the denominator *p*! represents the combination of *p* features in arbitrary ordering and the numerator |*S*| ! (*p* − |*S*| − 1)! denotes the combination of *p* features under a particular ordering after determining the subset S; $\frac{\left|S\right|!\left(p-\left|S\right|-1\right)!}{p!}$ is the proportion of feature combinations of subset *S*, and the sum of all possible subsets is equal to one.

The most obvious advantage of the Shapley value over traditional feature importance analysis is that it reflects the influence of the features in each sample and also shows the positive and negative nature of the influence. To capture the effects of pairwise interactions directly, we use Shapley interaction values, which ensure consistency while explaining the predicted local interaction effects [20].

10 $${\phi}_{i,j}=S\subseteq \setminus \left\{i,j\right\}\frac{\left|S\right|!\left(M-\left|S\right|-2\right)!}{2\left(M-1\right)!}{\delta}_{ij}(S)$$

11 $$, when\ i\ne j, and\ {\delta}_{ij}(S)={f}_x\left(S\cup \left\{i,j\right\}\right)-{f}_x\left(S\cup \left\{i\right\}\right)-{f}_x\left(S\cup \left\{j\right\}\right)+{f}_x(S)$$

Assuming that there are *N* samples with *M* features, the dimension of the Shapley value is *N* × *M*, whereas the dimension of the Shapley interaction values is *N* × *M* × *M*. That is, corresponding to one feature of a sample, the Shapley value consists of a single attribution value *ϕ*_*j*_, while Shapley interaction values are attributed by a series of interaction attribution values {*ϕ*_*i*1_, *ϕ*_*i*2_, ⋯, *ϕ*_*iM*_}. When the Shapley interaction value is greater than zero, the two variables generate a synergistic effect on vibrancy (i.e., a positive interaction), and vice versa have a negative interaction effect.

## Results

### Vibrancy of MSAs in Wuhan

**Fig. 3:**
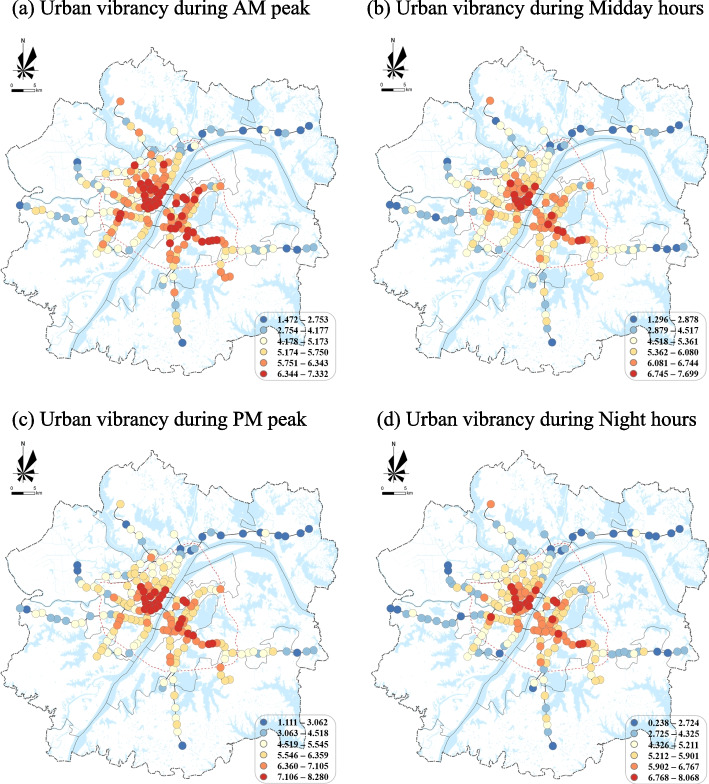
Urban vibrancy of MSAs during different times in Wuhan

Figure 3 displayed the spatial and temporal distribution of MSAs’ vibrancy during different times in Wuhan. In terms of time, people had relatively higher activity frequency and the highest agglomeration during PM peak, followed by the night and midday hours, and the lowest activity frequency and agglomeration during AM peak. Spatially, the distribution of MSAs’ vibrancy in Wuhan showed a clear center-edge structure, with the vibrancy inside the Third Ring Road significantly higher than outside. In addition, there was spatial heterogeneity in urban vibrancy among the three towns. Among them, Hankou old town on the north bank of the Yangtze River had the highest vibrancy, followed by Wuchang old town on the south bank. The results suggest that the degree of urban activity and crowd gathering was related to different types of time and space.

### Relative importance of built-environment features

We performed a covariance diagnosis on the independent variables. The variance inflation factors were all less than 10, proving that multicollinearity was not a problem. After that, we constructed the GBDT model and interpreted it by SHAP. The R-squared of the training and test sets of the model during four times was all above 0.75, indicating that our model was valid.

**Fig. 4:**
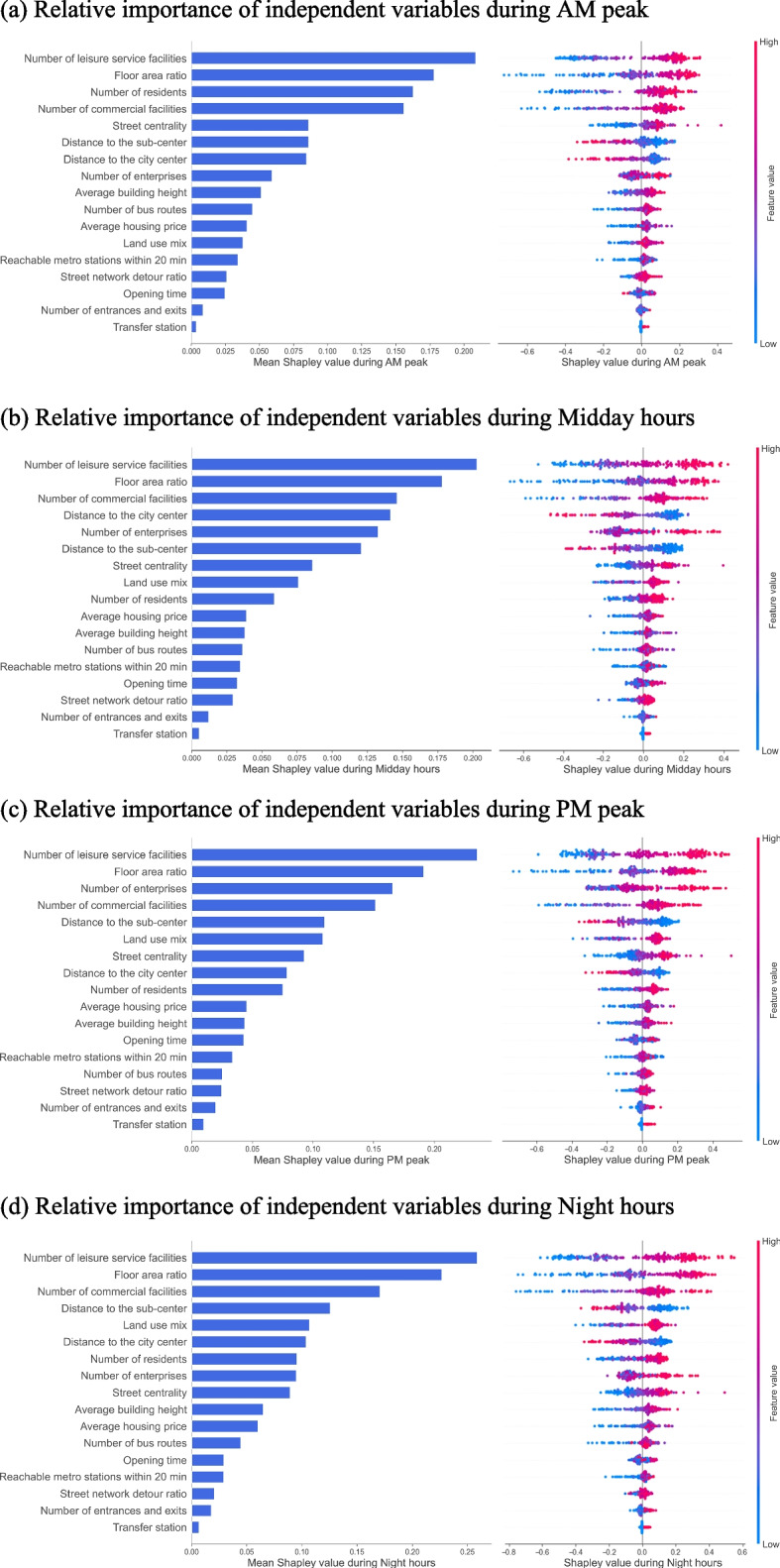
Relative importance of built-environment features during different times

As shown in Fig. 4, nine features contributed more than 0.1 during different times. Among them, the number of leisure facilities and FAR were the two most influential variables on vibrancy during all times, and the number of commercial facilities ranked third during midday and evening hours. These variables evenly changed predicted vibrancy by 0.26, 0.23, and 0.17, respectively, during night hours, which was significantly higher than during other times. The third highest during AM peak was the number of residents, with a contribution of 0.16, and the third highest during PM peak was the number of enterprises, with a contribution of 0.17. The mean Shapley values for the distance to the city center and sub-center were higher during midday hours at 0.14 and 0.12, respectively. Subsequently, the contribution of street centrality did not vary much across time, and the contribution of land use mix was higher during PM peak and night hours.

### Nonlinear effects of built-environment features

**Fig. 5:**
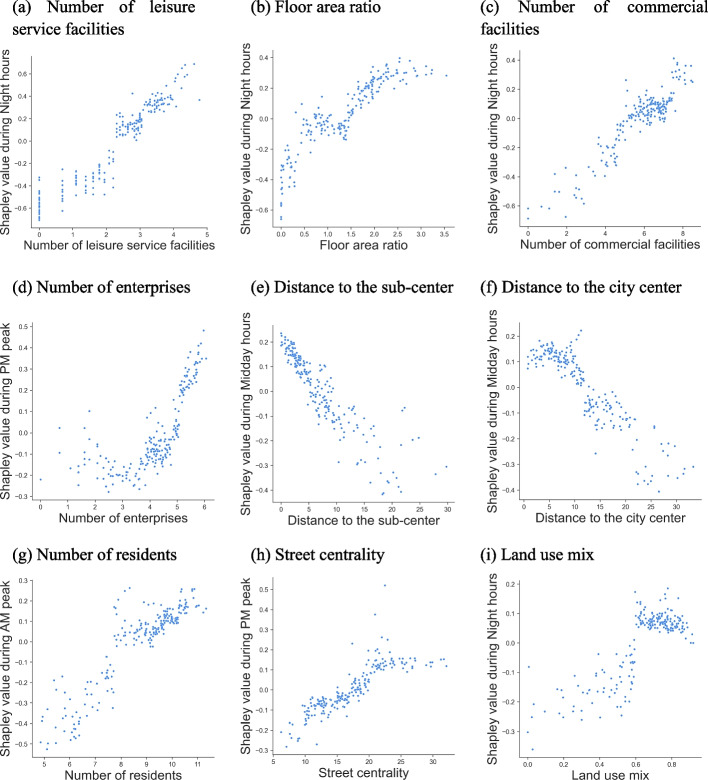
Local effects of built-environment features on the vibrancy

Figure 5 showed a partial dependency plot derived from the GBDT model, where we visualized the impact of the nine key independent variables on predicting MSAs’ vibrancy during the periods with the highest contribution. Overall, leisure facilities, commercial facilities, and the distance to the sub-center had approximately linear associations with urban vibrancy, while the others exhibited complex nonlinear effects. Specifically, the FAR reflected specific nonlinear and threshold effects on urban vibrancy, with the inhibitory effect on vibrancy gradually decreasing to no effect as it increased from 0 to 1.4. Then, the effect showed a positive correlation with an approximate logarithmic curve and stabilized as the FAR grew beyond 2.2.

The number of enterprises, distance to the city center, the number of residents, street centrality, and land use mix also showed diverse nonlinear correlations. The number of enterprises from 3.5 to 6 showed an approximately linear positive correlation with vibrancy. Besides, the distance to the city center had an upward trend in the 5km range (approximating Wuhan’s second ring road), after which it gradually decreased and dropped to a negative value in the area 12km away (approximating Wuhan’s third ring road), indicating that the further the distance outside the central city area had a greater inhibiting effect on vibrancy. Moreover, the number of residents showed a weak upward trend in the field from 8 to 11. Street centrality was positively correlated with vibrancy from 5 to 22, with no change in the local effect beyond 22. Similarly, the land use mix contributed to vibrancy after exceeding 0.6, with no change for further increases.

### Interaction effects among built-environment features

**Fig. 6:**
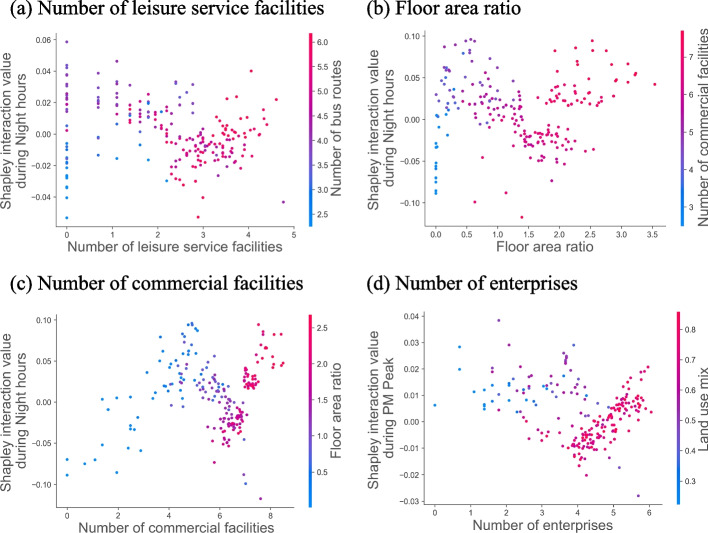
Local interaction effects among built-environment features

Our study further investigated the interaction among built-environment features. There were synergistic effects between leisure facilities and bus routes, commercial facilities and floor area ratio, and employment facilities and land use mix, as shown in Fig. 6. Leisure facilities and bus routes could generate synergistic effects, with a positive effect on vibrancy when the number of leisure facilities was less than three and the number of bus routes was in the range of 3–5, and when the number of leisure facilities was greater than three and the number of bus routes was greater than five. Similarly, there was an interaction between commercial facilities and FAR. For MSAs with FAR below 1.5, the interaction became larger as commercial facilities increased, and a synergistic effect was generated after the number exceeded 3.5. However, for MSAs with FAR above 1.5, the interaction was negative when the number of commercial facilities ranged from 5.5 to 6.8. Their interaction increased with increasing commercial facilities again when the number exceeded 6.8. Moreover, there was an interaction between enterprises and the land use mix, with their Shapley interaction value increasing with the number of enterprises when the land use mix was greater than 0.6, generating synergistic effects when the number of enterprises exceeded 5.

## Discussion

Improving urban vibrancy can facilitate human activities and interactions, enhance the attractiveness of urban spaces, and offer more easeful living conditions for residents [1, 33]. TOD is considered to promote urban vibrancy, yet in reality, its implementation does not always lead to vibrant daily life. To better respond to the decline in the vibrancy of public transportation areas since the epidemic, planners wanting to improve the vibrancy around metro stations must rethink how to optimize the built environment of metro station areas. From the above results, we obtain five main findings.

First, there was spatial-temporal variability in the MSAs’ vibrancy in Wuhan. The temporal differences were similar to the results of previous studies. For example, Wu et al. [34] showed that people’s activity frequency was relatively low during typical sleep and work time and relatively high during leisure and dinner time. Wang et al. [19] showed that crowd activity was most dispersed in the morning and then began to climb in intensity in these neighborhoods as workers and tourists flocked to the core. Spatially, the distribution of MSAs’ vibrancy in Wuhan showed a clear center-edge structure and spatial heterogeneity among the three towns, which was consistent with the study of Li et al. [4]. He found that urban vibrancy appeared to be much higher on the northern bank of the Yangtze River, where the old city of Hankou was located, and less active in the broader area at the city’s periphery.

Second, the relative importance of built-environment features influencing MSAs’ vibrancy indicates that leisure facilities, FAR, commercial facilities, and employment facilities were the most dominant explanatory variables. The results for leisure, commercial, and employment facilities were consistent with existing studies. Li et al. [4] found that shopping and leisure density were positive factors that induced urban vibrancy. In addition, Wu et al. [35] showed that industries POI and business POI were the largest contributing factors influencing urban vibrancy. That is probably because to revitalize a place, it is necessary to provide as many services as possible and create opportunities for people to enjoy various daily activities. However, the importance of FAR was inconsistent with the spatiotemporal vibrancy study in Shenzhen [22], where the floor area was not a good predictor. The difference may be because the analysis in this study was based on the vibrancy of metro station areas, and some studies have found that the effect of floor area on vibrancy varies depending on the distance to the nearest metro station [11].

Third, there were significant differences in ranking the effects of built-environment features on MSAs’ vibrancy during different times. Leisure facilities, FAR, and commercial facilities contributed remarkably more at night than at other periods. The land use mix was also relatively more important at night, which was in line with the findings of previous studies [13, 34]. They showed that consumption POI, land use mix, and FAR were more influential at night. This suggests that people tend to engage in diverse activities at night, especially leisure and business-related activities and that buildings can provide space for intensive and continuous activities. Besides, the contribution of enterprises was significantly higher during PM peak, while that of residents was higher during AM peak, which was different from the findings of previous studies on vibrancy. Chen et al. [22] showed that population density contributed more to urban vibrancy in the evening and that company POI had a weaker temporal association with vibrancy from 10:00 to 22: 00 on weekdays. It may be because the vibrancy around metro stations would be more associated with the commuting behavior of residents on weekdays, with people leaving from their residential neighborhoods during AM peak and leaving from work during PM peak [25, 36].

Fourth, there were approximately linear or complex nonlinear relationships between the built-environment features and MSAs’ vibrancy. Leisure and commercial facilities showed an approximately positive linear relationship, the distance to the sub-center showed a negative linear association, and most of the built-environment features had complex nonlinear effects. For example, the nonlinear pattern of FAR was similar to the relationship between building form and vibrancy in Shenzhen [20]. It can be considered that buildings are the carriers of various functional services and facilities. Low building density substantially inhibits vibrancy. After the FAR exceeds 1.4, vibrancy improves with the increase of the index. Still, once the building demands have been generally satisfied, further improvements of FAR for vibrancy promotion will be less effective and even ineffective. The number of enterprises needed more than 3.5 to show a positive correlation with vibrancy. One explanation is that fewer enterprises have little effect on vibrancy, while with enough enterprises, the MSAs are employment-oriented making vibrancy greater as enterprises increase. Besides, the nonlinear pattern of street centrality was also consistent with the results of previous studies. For example, Yang et al. [8] found that street density boosts vibrancy, saturating after reaching 32,000 m/km^2^. It can be assumed that well-connected streets can attract pedestrians; however, once the demand for access is generally satisfied, the effect of further increasing street centrality to promote vibrancy will be reduced or even ineffective. Moreover, the local effect of the land use mix was similar to the results of a study on ridership in Washington [37]. This may be because higher land use mix implies more diverse and rich activities in the area, which is conducive to attracting people [38], while after reaching 0.6, the relationship between various activities is balanced and the promotion of vibrancy is at its highest.

Finally, some features produced synergistic effects when certain conditions were met. The study found that multimodal transportation was more effective at leisure-dominated stations, high-density development was more effective at commercial-dominated stations, and mixed development was more effective at employment-oriented stations. Fewer leisure facilities and medium bus routes, and more leisure facilities and bus routes generated synergistic effects, while fewer recreational facilities and more bus routes had adverse interaction effects. This might be because MSAs with fewer leisure facilities lack attractiveness to residents, and increased bus routes can drive regional traffic to and from the area. However, when transit is abundant, a convenient external transportation system may promote outbound travel and reduce vibrancy, consistent with previous research findings [39]. With sufficient leisure facilities, well-developed public transport will drive residents outside the area for activities. Similarly, there were synergies between low FAR and medium commercial facilities, and between high FAR and sufficient commercial facilities, while the interaction value between high FAR and medium commercial facilities was negative. It can be argued that, for low-density MSAs, increased commercial services trigger more economic activities. However, the increase may take space away from commercial-dominant activities for high-density station areas. Yet when there are enough commercial facilities, MSAs become commercial-dominated, allowing synergies to increase, which is consistent with the findings of Guangzhou’s exploration of community vibrancy [40]. Additionally, high land-use mix and sufficient enterprises generated synergies. This is probably because the increase of enterprises in mixed development areas will employ more people, creating vibrancy.

These findings have important planning and policy implications for promoting the restoration of urban vibrancy around metro stations since the epidemic. First, to make more efficient use of limited resources, priority is given to improving the MSAs in leisure facilities, floor area ratio, commercial facilities, and employment facilities. Second, the relative importance during different times indicates the different mechanisms of influence on the vibrancy of TOD areas. Based on prioritizing the provision of leisure services and balancing building density, it is more helpful to facilitate the recovery of MSAs’ vibrancy by conforming to the national policy to activate nighttime businesses and markets vigorously and by improving the arrangement related to community residents during AM peak and the attributes related to employment facilities during PM peak. Third, the nonlinear relationships between built-environment features and vibrancy provide a range of effective intervals to promote MSAs’ vibrancy. In planning practice, leisure, commercial, and employment services can be improved toward areas with higher negative Shapley values, and the FAR should be set from 1.4 to 2.2 to accelerate vibrancy best. Finally, the identified synergies suggest that planning TOD to facilitate vibrancy should focus on multiple dimensions. In the future, it would be possible to develop rail stations by combining a leisure-led approach with multimodal transportation, a commercially led approach with high-density development, and an employment-oriented approach with mixed development.

However, there are some limitations to this study. First, we selected data with temporal variability to measure the vibrancy. Two of these data sources are only available from smartphone users, which may cause the measure to be less comprehensive. Further research should include non-smartphone users and find available data from more dimensions, such as economy and culture, or through questionnaires to obtain residents’ feelings. Second, only the vibrancy data change over time, whereas the variables of TOD remain the same. More detailed temporal factors, such as the opening hours of leisure and commercial facilities, should be considered. Finally, this study selected four times on weekdays in 2021 for comparison. Further research could delve into the temporal and spatial changes in human behavior.

## Conclusions

This study examines the nonlinear and synergistic effects of the built-environment features on urban vibrancy during different times at the TOD level in Wuhan through the GBDT and SHAP explanatory models. We systematically constructed the built-environment features of TOD based on the 5D framework and measured an integrated vibrancy index based on multiple big data sources across specified periods. The study shows differences in the effects of built-environment features on the MSAs’ vibrancy over time. Combined rankings during different times show that the most important contributors to the MSAs’ vibrancy are a higher number of leisure, commercial, and employment facilities and high FAR. Moreover, we found that there were approximately linear or complex nonlinear relationships between the built-environment features and the MSAs’ vibrancy, and synergistic effects among some features.

Our study evaluated the comprehensive vibrancy of metro station areas over time and built a framework of explanatory variables affecting the vibrancy from the perspective of the built environment. We described the nonlinear effects of built-environment features on the MSAs’ vibrancy and proposed planning suggestions related to the organization form of rail transport. It promoted the city’s balanced, healthy, and sustainable development from the perspective of urban planning. Meanwhile, it provided a reliable theoretical framework for future academic research on the influence of built-environment features on MSAs’ vibrancy.

## Supplementary Information

**Additional file 1: **Literature review. **Table S1.** Recent studies incorporating diverse proxies for urban vibrancy.

## Data Availability

The data that support the findings of this study are available from Wuhan Transportation Development Strategy Institute but restrictions apply to the availability of these data, which were used under license for the current study, and so are not publicly available. Data are however available from the authors upon reasonable request and with permission of Wuhan Transportation Development Strategy Institute.

## Abbreviations

TOD: Transit-oriented development
GBDT: Gradient boosting decision tree
MSAs: Metro station areas
SHAP: Shapley additive explanations
