# Supplementary material for: Understanding nonlinear and synergistic effects of the built environment on urban vibrancy in metro station areas
Source: J. Eng. Appl. Sci. 2023 Mar 9;70(1):18. doi: 10.1186/s44147-023-00182-z (PMC9995723; doi:10.1186/s44147-023-00182-z)
Supplement: Supplementary file 1 — Additional file 1: Literature review. Table S1. Recent studies incorporating diverse proxies for urban vibrancy. [file 44147_2023_182_MOESM1_ESM.docx]

**Additional file 1. Literature review**

***Description of urban vibrancy***

Urban vibrancy was first introduced by JacobsJacobs(1961), who claimed that "liveness and variety attract more liveness; deadness and monopoly repel life." She stressed that "liveness" was bred by the presence of pedestrians in streets at any time of the day, whereas the loss of pedestrian activities led to " deadness." Until now, the definition of urban vibrancy can be divided into two categories. Some depict urban vibrancy in terms of the presence of people in streets and public spaces, the synergy among all kinds of people, and various human activities and opportunities [2,3]; whereas others define vibrancy as the character of urban spaces to accommodate diverse functions [4,5]. Although the descriptions of urban vibrancy vary, the basic consensus is that urban vibrancy constitutes a well-designed urban environment that stimulates human interaction and activities [6].

With the continuous development of information technology, big data and open data are becoming increasingly popular, providing a solid foundation for accurate assessment of city vibrancy assessment [7]. For example, data such as Tencent location data and Baidu heat index can record pedestrian density in different spaces and times [8,9], which directly reflects people's movement and clustering and is considered a valid representation of urban vibrancy [10]. At the TOD level, compact, mixed TOD-oriented development and efficient, high-quality public transportation can increase the probability of active travel by residents and trigger more social and economic activity [11]. Metro ridership can truly record people's mobility [12]. In addition, studies have shown that social media check-in data can provide an intensity of social activity that transcends physical limitations [13,14]. The number of check-ins at a location reflects the popularity of the area and the degree of attracting users' attention [15], which can reflect a city's population distribution, mobility, and residents' travel preferences, and better reflect the spatial-temporal characteristics of urban vibrancy[16]. Further, the combined vibrancy of multiple big data sources can convey standard information across numerous indicators and exclude random variations and biases that can be associated with single data, which is more beneficial to accurately characterize urban vibrancy [17,18].

Table S1. Recent studies incorporating diverse proxies for urban vibrancy.

| Focus | Feature | Source | Literature | Proxy | Data source | Study area and scale |
| --- | --- | --- | --- | --- | --- | --- |
| Human activity | Single proxy | Survey data | Mouratidis & Poortinga [19] | The extent to which a place feels alive or lively | The population-based survey | Neighborhoods in Oslo metropolitan area |
|  |  |  | Mu et al.[20] | Diversity analysis of age group, visitor density per hour, space usage intensity in peak time, the richness of activity type, and the behavior map | Observations of visitors | Three old community parks in Zhengzhou |
|  |  |  | Delclòs-Alió et al.[21] | Daily walking itinerary | A smartphone tracking experiment | The BMR |
|  |  | Big data | Kim [22] | The virtual dimension of urban vitality | Wi-Fi hotspots | Six administrative districts of Seoul |
|  |  |  | M. Li et al.[7] | Pedestrian flow | Citygrid sensors | Twenty-three street segments of Baitasi Area in Beijing |
|  |  |  | L. Chen et al.[6] | Tencent location data | Tencent location-based mobile phone applications | Shenzhen, China |
|  |  |  | Yang et al.[9] | Baidu Heat Index | Baidu Heat Map | Six districts with subway stations in Shenzhen |
|  |  |  | Sulis et al.[12] | Twitter data | Twitter API | Areas assigning bus stops that fall within  the 400 m range to the correspondent station in London |
|  |  |  | Meng & Xing [13] | Review data | Social media, such as Yelp and Google Maps | Futian district, Shenzhen, Guangdong Province |
|  |  |  | C. Wu et al.[16] | Check-in data | Social media Sina Visitor System | Shenzhen, China |
|  | Comprehensive proxy |  | Li et al. [17] | Comprehensive index (Street vitality+ Weibo check-ins) | Baidu Map; SINA | 1025 community blocks in Wuhan |
|  |  |  | Tu et al. [18] | Comprehensive vibrancy index (Density of POI+ Density of social media check-ins+ Density of mobile phone records) | POIs; social media check-ins; Mobile phone data | 491 traffic analysis zones in Shenzhen |
|  |  |  | Xiao et al.[23] | Comprehensive index（ metro ridership+ Resident density+ social activity intensity+ economic activity intensity） | Metro smartcard data; Sina Weibo check-in data; Dianping review data; Baidu positioning record data | 166 metro stations in Shenzhen |
|  |  |  | P. Zeng et al. [24] | Comprehensive index（the bicycle-share ridership +spatial distance + spatial diversity） | Bicycle-Sharing Data | 98 subdistricts in Shanghai |
|  |  |  | Huang et al. [10] | Comprehensive index（ Social activity intensity+ Economic activity intensity + Pedestrian density） | Mobile phone GPS positioning requests, Sina Weibo data and Dianping life services reviews | Shanghai, China |
| Urban space | Single proxy |  | Lan et al.[25] | Night-time light | Night-time light data | 35 large- and medium-sized cities in China |
|  |  |  | Wu et al.[26] | Geo-tagged small food facilities | Point of interest data | Twelve cities in China |
|  |  |  | Chen et al. [27] | The number of cafes | Qichacha | Cafes in the GBA |
|  | Comprehensive proxy |  | Zeng et al. [28] | Comprehensive index (density, livability, accessibility and diversity) | Google and Baidu API | Communities in Chicago US and Wuhan China |

***Urban vibrancy and built-environment features of TOD***

TOD densifies station areas, provides a mix of residential, recreational, commercial, and office uses, and reduces walking distances between destinations by implementing shorter streets [9], which can make street life vibrant. Existing studies describe the built-environment features of TOD in terms of density, diversity, design, destination accessibility, and distance to transit (5Ds) [6,29].

To elaborate, high-density (both population and spatial density) environments are closely associated with maintaining urban vibrancy [1]. The concentration of the population further contributes to the vibrancy and encourages face-to-face human interaction [10]. The type and density of buildings are also emphasized as an important physical basis for urban vibrancy [30]. Contrastingly, diversity is mainly reflected in the mix of land uses. This makes neighborhoods functional and attracts various human activities by enabling complementary activities such as leisure, dining, and shopping [31]. However, overcrowding, high density, and diversity can cause psychological stress, which can negatively affect urban vibrancy [32]. Concerning design, which refers to street design, highly connected streets increase neighborhood accessibility, thereby enhancing district vibrancy [33,34]. Distance to urban centers is used as a measure of regional accessibility, and studies have found that urban vibrancy decreases from the central city to the periphery [35]. Furthermore, multimodal transportation is also an important factor affecting urban vibrancy. More bus routes around a metro station indicate a stronger regional transit capacity, which is more conducive to attracting transit traffic and thus has a positive impact on the surrounding regional vibrancy [23].

It has also been shown that the characteristics of subway stations can affect pedestrian density in TOD areas. Higher connectivity, more entrances and exits, and operation times of metros, and transfer stations attract more riders [36,37]. Besides the built environment, socioeconomic factors are important for daily activities, as indicated by housing prices being positively correlated with urban vibrancy [10].

Several studies focusing on temporal differences in the relationship between the built-environment features and urban vibrancy have emerged in recent years. It is more common to compare differences between weekdays and weekends [38]. A study in Guangzhou showed that high-rise commercial buildings dictated community vibrancy on weekdays, while public services with large floor areas had the greatest impact on community vibrancy on weekends [39]. Other studies have focused on diurnal differences [40,41]. Yang et al (2021) verified that most built environment variables have similar trends on urban vibrancy during the day and night, while the share of office area had a greater diurnal effect. What’s more, studies are looking at urban vibrancy during different times of the day, suggesting that different built-environment features may have different spatial-temporal relationships with human activity intensity [6,16]. However, existing studies on the relationship between the built environment and the vibrancy in public transportation areas are biased towards comparing spatial differences [23], and less temporal variability has been introduced for comprehensive studies. Previous studies have demonstrated that human activities in public transportation areas are significantly influenced by built-environment features over time [29,42]. Therefore, it is necessary to explore the relationship between the built-environment features and the vibrancy around metro stations during different times.

***Research methods***

In most of the existing studies on the built environment and urban vibrancy, a linear or generalized linear relationship between the two has been assumed [10,17]. These studies provide a solid basis for understanding the relationship between the built-environment features and urban vibrancy but ignore the possible nonlinear and synergistic effects of the two [43]. In recent years, scholars have gradually adopted ML techniques to elucidate the more refined nonlinear statistical relationship between them. Yang et al (2021) used the GBDT model to demonstrate that there was a general nonlinear threshold effect of the built-environment features on urban vibrancy. For example, in the interval of 0–1.50 million m^2^, the total floor area had a positive and approximately linear association with urban vibrancy, and beyond 1.50 million m^2^, the vibrancy rose substantially and was saturated at about 1.65 million m^2^. In addition, ML can determine the relative importance of independent variables, which is efficient for planners to prioritize their decisions within limited conditions. Based on a study in Shenzhen, it was found that the features that most influence urban vibrancy during the day and night are both business services and industries, except that they differ in the order of importance during both periods [41].

Moreover, ML can also verify synergistic effects among the effects of explanatory variables, as the effects of the built-environment features of TOD on vibrancy may be moderated by third-party variables. However, ML algorithms lack local interpretation of individual units, resulting in a “black-box” predictive model with unintelligible interpretability [44]. And the SHAP, proposed by Lundberg & Lee(2017), can overcome this property by providing consistent interpretability inspired by cooperative game theory. For example, Xiao et al. (2021) used the GBDT and SHAP explanatory models to provide "Shapley interaction values" to show that different dimensional variables generate synergistic effects when certain conditions are met. For example, local effects are amplified when the functional mixture is greater than 0.8, and the distance to the central business district (CBD) is above 1 km. Therefore, the GBDT approach, which is a more advanced ML model, is selected to explore the relationship between the built-environment features and urban vibrancy and is explained by SHAP in this study.

***Research gaps***

Despite the numerous studies on urban vibrancy, there are still some research gaps. First, in the existing studies on the built-environment features and urban vibrancy in public transportation areas [23], there is a bias toward comparing spatial differences and less focus on temporal differences. Second, most studies assume a linear or generalized linear relationship between the built-environment features and urban vibrancy, but in fact, they are generally nonlinearly related. Finally, the effects of individual built-environment features on vibrancy may be moderated by third parties. Identifying the interactions among variables can help planners capture complex relationships [31,46].

**References**

[1] J. Jacobs, The Death and Life of Great American Cities, Vintage, 1961.

[2] P.R. Maas, Towards a theory of urban vitality, (1984).

[3] J. Montgomery, Making a city: Urbanity, vitality and urban design, J. Urban Des. 3 (1998) 93–116.

[4] J. Gehl, Life between buildings, New York: Van Nostrand Reinhold, 1987.

[5] I. Bentley, Responsive environments: A manual for designers, Archit. Pr. (1985).

[6] L. Chen, L. Zhao, Y. Xiao, Y. Lu, Investigating the spatiotemporal pattern between the built environment and urban vibrancy using big data in Shenzhen , China, Comput. Environ. Urban Syst. 95 (2022). https://doi.org/10.1016/j.compenvurbsys.2022.101827.

[7] M. Li, J. Liu, Y. Lin, L. Xiao, J. Zhou, Revitalizing historic districts: Identifying built environment predictors for street vibrancy based on urban sensor data, Cities. 117 (2021) 103305. https://doi.org/10.1016/j.cities.2021.103305.

[8] S. Liu, S.Q. Lai, C. Liu, L. Jiang, What influenced the vitality of the waterfront open space? A case study of Huangpu River in Shanghai, China, Cities. 114 (2021) 103197. https://doi.org/10.1016/j.cities.2021.103197.

[9] J. Yang, J. Cao, Y. Zhou, Elaborating non-linear associations and synergies of subway access and land uses with urban vitality in Shenzhen, Transp. Res. Part A Policy Pract. 144 (2021) 74–88. https://doi.org/10.1016/j.tra.2020.11.014.

[10] B. Huang, Y. Zhou, Z. Li, Y. Song, J. Cai, W. Tu, Evaluating and characterizing urban vibrancy using spatial big data: Shanghai as a case study, Environ. Plan. B Urban Anal. City Sci. 47 (2020) 1543–1559. https://doi.org/10.1177/2399808319828730.

[11] L. Xiao, S. Lo, J. Zhou, J. Liu, L. Yang, Predicting vibrancy of metro station areas considering spatial relationships through graph convolutional neural networks: The case of Shenzhen, China, Environ. Plan. B Urban Anal. City Sci. 48 (2021) 2363–2384. https://doi.org/10.1177/2399808320977866.

[12] P. Sulis, E. Manley, C. Zhong, M. Batty, Using mobility data as proxy for measuring urban vitality, J. Spat. Inf. Sci. 16 (2018) 137–162. https://doi.org/10.5311/JOSIS.2018.16.384.

[13] Y. Meng, H. Xing, Exploring the relationship between landscape characteristics and urban vibrancy: A case study using morphology and review data, Cities. 95 (2019) 102389. https://doi.org/10.1016/j.cities.2019.102389.

[14] J. Ouyang, H. Fan, L. Wang, D. Zhu, M. Yang, Revealing urban vibrancy stability based on human activity time-series, Sustain. Cities Soc. 85 (2022) 104053. https://doi.org/10.1016/j.scs.2022.104053.

[15] Q. He, W. He, Y. Song, J. Wu, C. Yin, Y. Mou, The impact of urban growth patterns on urban vitality in newly built-up areas based on an association rules analysis using geographical ‘big data,’ Land Use Policy. 78 (2018) 726–738. https://doi.org/10.1016/j.landusepol.2018.07.020.

[16] C. Wu, X. Ye, F. Ren, Q. Du, Check-in behaviour and spatio-temporal vibrancy: An exploratory analysis in Shenzhen, China, Cities. 77 (2018) 104–116. https://doi.org/10.1016/j.cities.2018.01.017.

[17] X. Li, Y. Li, T. Jia, L. Zhou, I.H. Hijazi, The six dimensions of built environment on urban vitality: Fusion evidence from multi-source data, Cities. (2021) 103482. https://doi.org/10.1016/j.cities.2021.103482.

[18] W. Tu, T. Zhu, J. Xia, Y. Zhou, Y. Lai, J. Jiang, Q. Li, Portraying the spatial dynamics of urban vibrancy using multisource urban big data, Comput. Environ. Urban Syst. 80 (2020) 101428. https://doi.org/10.1016/j.compenvurbsys.2019.101428.

[19] K. Mouratidis, W. Poortinga, Built environment, urban vitality and social cohesion: Do vibrant neighborhoods foster strong communities?, Landsc. Urban Plan. 204 (2020) 103951. https://doi.org/10.1016/j.landurbplan.2020.103951.

[20] B. Mu, C. Liu, T. Mu, X. Xu, G. Tian, Y. Zhang, G. Kim, Spatiotemporal fluctuations in urban park spatial vitality determined by on-site observation and behavior mapping: A case study of three parks in Zhengzhou City, China, Urban For. Urban Green. 64 (2021). https://doi.org/10.1016/j.ufug.2021.127246.

[21] X. Delclòs-Alió, A. Gutiérrez, C. Miralles-Guasch, The urban vitality conditions of Jane Jacobs in Barcelona: Residential and smartphone-based tracking measurements of the built environment in a Mediterranean metropolis, Cities. 86 (2019) 220–228. https://doi.org/10.1016/j.cities.2018.09.021.

[22] Y.L. Kim, Seoul’s Wi-Fi hotspots: Wi-Fi access points as an indicator of urban vitality, Comput. Environ. Urban Syst. 72 (2018) 13–24. https://doi.org/10.1016/j.compenvurbsys.2018.06.004.

[23] L. Xiao, S. Lo, J. Liu, J. Zhou, Q. Li, Nonlinear and synergistic effects of TOD on urban vibrancy: Applying local explanations for gradient boosting decision tree, Sustain. Cities Soc. 72 (2021) 103063. https://doi.org/10.1016/j.scs.2021.103063.

[24] P. Zeng, M. Wei, X. Liu, Investigating the spatiotemporal dynamics of urban vitality using bicycle-sharing data, Sustain. 12 (2020). https://doi.org/10.3390/su12051714.

[25] F. Lan, X. Gong, H. Da, H. Wen, How do population inflow and social infrastructure affect urban vitality? Evidence from 35 large- and medium-sized cities in China, Cities. 100 (2020) 102454. https://doi.org/10.1016/j.cities.2019.102454.

[26] J. Wu, Y. Lu, H. Gao, M. Wang, Cultivating historical heritage area vitality using urban morphology approach based on big data and machine learning, Comput. Environ. Urban Syst. 91 (2022) 101716. https://doi.org/10.1016/j.compenvurbsys.2021.101716.

[27] Z. Chen, B. Dong, Q. Pei, Z. Zhang, The impacts of urban vitality and urban density on innovation: Evidence from China’s Greater Bay Area, Habitat Int. 119 (2022) 102490. https://doi.org/10.1016/j.habitatint.2021.102490.

[28] C. Zeng, Y. Song, Q. He, F. Shen, Spatially explicit assessment on urban vitality: Case studies in Chicago and Wuhan, Sustain. Cities Soc. 40 (2018) 296–306. https://doi.org/10.1016/j.scs.2018.04.021.

[29] J. Zhou, Y. Yang, P. Gu, L. Yin, F. Zhang, F. Zhang, D. Li, Can TODness improve (expected) performances of TODs? An exploration facilitated by non-traditional data, Transp. Res. Part D Transp. Environ. 74 (2019) 28–47. https://doi.org/10.1016/j.trd.2019.07.008.

[30] Y. Ye, D. Li, X. Liu, How block density and typology affect urban vitality: an exploratory analysis in Shenzhen, China, Urban Geogr. 39 (2018) 631–652. https://doi.org/10.1080/02723638.2017.1381536.

[31] Q. Shao, W. Zhang, X. Cao, J. Yang, J. Yin, Threshold and moderating effects of land use on metro ridership in Shenzhen: Implications for TOD planning, J. Transp. Geogr. 89 (2020) 102878. https://doi.org/10.1016/j.jtrangeo.2020.102878.

[32] G. Simmel, The metropolis and mental life, in: Urban Sociol. Read., Routledge, 2012: pp. 37–45.

[33] S. Monajem, F. Ekram Nosratian, The evaluation of the spatial integration of station areas via the node place model; an application to subway station areas in Tehran, Transp. Res. Part D Transp. Environ. 40 (2015) 14–27. https://doi.org/10.1016/j.trd.2015.07.009.

[34] A. Dhanani, L. Tarkhanyan, L. Vaughan, Estimating pedestrian demand for active transport evaluation and planning, Transp. Res. Part A Policy Pract. 103 (2017) 54–69. https://doi.org/10.1016/j.tra.2017.05.020.

[35] W. Yue, Y. Chen, Q. Zhang, Y. Liu, Spatial explicit assessment of urban vitality using multi-source data: A case of Shanghai, China, Sustain. 11 (2019). https://doi.org/10.3390/su11030638.

[36] S. Li, D. Lyu, X. Liu, Z. Tan, F. Gao, G. Huang, Z. Wu, The varying patterns of rail transit ridership and their relationships with fine-scale built environment factors: Big data analytics from Guangzhou, Cities. 99 (2020) 102580. https://doi.org/10.1016/j.cities.2019.102580.

[37] S. Su, H. Zhang, M. Wang, M. Weng, M. Kang, Transit-oriented development (TOD) typologies around metro station areas in urban China: A comparative analysis of five typical megacities for planning implications, J. Transp. Geogr. 90 (2021) 102939. https://doi.org/10.1016/j.jtrangeo.2020.102939.

[38] S. hyeok Lee, J.E. Kang, Impact of particulate matter and urban spatial characteristics on urban vitality using spatiotemporal big data, Cities. 131 (2022) 104030. https://doi.org/10.1016/j.cities.2022.104030.

[39] N. Niu, L. Li, X. Li, J. He, The structural dimensions and community vibrancy: An exploratory analysis in Guangzhou, China, Cities. 127 (2022). https://doi.org/10.1016/j.cities.2022.103771.

[40] C. Xia, A.G.O. Yeh, A. Zhang, Analyzing spatial relationships between urban land use intensity and urban vitality at street block level: A case study of five Chinese megacities, Landsc. Urban Plan. 193 (2020) 103669. https://doi.org/10.1016/j.landurbplan.2019.103669.

[41] C. Wu, Y. Ye, F. Gao, X. Ye, Using street view images to examine the association between human perceptions of locale and urban vitality in Shenzhen , China, Sustain. Cities Soc. J. 88 (2023). https://doi.org/10.1016/j.scs.2022.104291.

[42] Z. Gan, M. Yang, T. Feng, H.J.P. Timmermans, Examining the relationship between built environment and metro ridership at station-to-station level, Transp. Res. Part D Transp. Environ. 82 (2020) 102332. https://doi.org/10.1016/j.trd.2020.102332.

[43] H. Bi, Z. Ye, H. Zhu, Examining the nonlinear impacts of built environment on ridesourcing usage : Focus on the critical urban sub-regions, J. Clean. Prod. 350 (2022) 131314. https://doi.org/10.1016/j.jclepro.2022.131314.

[44] S. Su, Z. Wang, B. Li, M. Kang, Deciphering the influence of TOD on metro ridership: An integrated approach of extended node-place model and interpretable machine learning with planning implications, J. Transp. Geogr. 104 (2022) 103455. https://doi.org/10.1016/j.jtrangeo.2022.103455.

[45] S.M. Lundberg, S.I. Lee, A unified approach to interpreting model predictions, Adv. Neural Inf. Process. Syst. 2017-Decem (2017) 4766–4775.

[46] C. Ding, X. Cao, Y. Wang, Synergistic effects of the built environment and commuting programs on commute mode choice, Transp. Res. Part A Policy Pract. 118 (2018) 104–118. https://doi.org/10.1016/j.tra.2018.08.041.
